# An integrated genomic and phenotypic insight into safety performance and probiotic potential of a novel strain *Pediococcus pentosaceus* K6 derived from Tibetan kefir

**DOI:** 10.3389/fmicb.2026.1894704

**Published:** 2026-07-23

**Authors:** Xiaochen Huang, Yingying Yang, Xinhe Zhang, Wanming Wang, Qunfa Yun, Saiqin Guo, Shuqi Guo, Jiayi Ge, Zhina Chen

**Affiliations:** 1 School of Food and Pharmaceutical Engineering, Zhaoqing University, Zhaoqing, China; 2 School of Biological Engineering, Huainan Normal University, Huainan, China; 3 Eco-Industrial Innovation Institute ZJUT, Quzhou, China

**Keywords:** genomic analysis, *Pediococcus pentosaceus*, probiotic characterization, safety assessment, Tibetan kefir

## Abstract

*Pediococcus pentosaceus* (*P. pentosaceus*) is a ubiquitous lactic acid bacterium recognized for enhancing the sensory properties, dietary benefits, and safety of fermented foods. This study isolates and characterizes a Tibetan kefir-derived strain, *P. pentosaceus* K6, using integrated genomic and phenotypic approaches. Whole-genome sequencing and *in vitro* assays confirmed a favorable safety profile for K6, showing no hemolytic activity or harmful metabolite production, and lacking pathogenic virulence determinants, transferable antibiotic resistance genes, or functional plasmid conjugation elements. K6 showed strain-specific probiotic features, including 83.83% survival at pH 3.0 for 2 h, 71.16% survival under simulated gastrointestinal digestion, 89.30% auto-aggregation at 48 h, and strong inhibition against *Listeria monocytogenes* (29.83 ± 0.06 mm). Compared with previously reported *P. pentosaceus* strains, K6 uniquely combines gastrointestinal tolerance, adhesion-related traits, and anti-*Listeria* activity. The antiSMASH analysis identified putative type III polyketide synthase and terpene gene clusters, providing genetic candidates for this antimicrobial phenotype. Furthermore, specific carbohydrate-active enzymes and exopolysaccharide biosynthetic genes indicate strong potential for carbohydrate metabolism and food matrix adaptation. Collectively, these genomic and phenotypic findings support *P. pentosaceus* K6 as a highly promising candidate for probiotic and biopreservation applications.

## 1 Introduction

Probiotics are a class of beneficial microorganisms that offer health benefits to the hosts upon sufficient intake. In recent years, mounting evidence regarding the health effects of intestinal flora has driven increased demand for probiotics (Lei et al., 2025). As a large and important category of probiotic family, lactic acid bacteria (LAB) are widely used in food production and health-related applications because of their remarkable effect on balancing intestinal flora, supporting immune function, and inhibiting pathogenic bacteria (Abedin et al., 2024). Among them, *Pediococcus pentosaceus* (*P. pentosaceus*) has obtained increasing scientific interest as it not only plays a key role in improving food quality but has also become a new promising probiotic with a variety of biological activity such as anti-inflammatory, anti-tumor, antioxidant, antibacterial, detoxification, and cholesterol-lowering (Jiang et al., 2021; Qi et al., 2021).

However, it is worth noting that the functional characteristics of probiotics show significant strain specificity, which means that taxonomic similarity does not guarantee functional equivalence, and even homogeneous strains from different sources may present completely different metabolic characteristics, environmental adaptability and host interactions (Jiang et al., 2021). Kefir is a traditional fermented milk drink from the Caucasus area, which contains a complex and diverse microbial ecosystem (Chen et al., 2022; Cheng et al., 2024). Studies indicate that LAB isolated from such a long-term naturally domesticated environment often possess natural resistance, unique fermentation performance (Chen et al., 2025) and excellent probiotic properties (Tan et al., 2022). Doğan and Ay (2021) reported that a strain of *P. pentosaceus* isolated from Turkish kefir exhibited high acid tolerance, bile salt resistance and hydrophobicity. Another strain *P. pentosaceus* SP2 originating from Greek kefir grains, displayed robust adherence to human colon cancer cells and markedly suppressed their proliferation (Mantzourani et al., 2019). The kombucha-sourced strain *P. pentosaceus* L3 demonstrated strong tolerance to the gastrointestinal environment, remarkable broad-spectrum antimicrobial and antifungal effects, as well as high viability during freeze-drying (Diguță et al., 2020). Given their excellent resistance, antibacterial efficacy and beneficial effects, screening for new strains from unique sources like kefir is crucial for enriching probiotic diversity.

Despite the broad application prospects of *P. pentosaceus*, strict safety assessment is still an indispensable prerequisite before utilization. It is reported that some strains in the genus *Pediococcus* may carry antibiotic-resistance genes, posing potential safety concerns (Mgomi et al., 2022). Traditional probiotic screening methods based on physicochemical experiments are simple and reliable (Aminu et al., 2024), however, they can neither elucidate the underlying molecular mechanisms, nor accurately predict the stability of strains or their potential pathogenic risks (Haranahalli Nataraj et al., 2024). Therefore, *in vitro* physicochemical characterization alone is far from sufficient to adequately assess their probiotic properties or rule out potential pathogenicity.

Over the past two decades, the rapid advancement of whole-genome sequencing (WGS) technology has provided a revolutionary tool for the accurate mining and evaluation of probiotic microbes (Sabino et al., 2025). WGS technology enables genomic analysis of probiotics, facilitating both the comprehensive screening for potential virulence factors and antibiotic resistance genes, and the elucidation of metabolic pathways associated with probiotic functions (Peng et al., 2023). Meanwhile, traditional *in vitro* physiological and biochemical assays can provide definitive phenotypic experimental validation. Consequently, integrating these two approaches for a multi-dimensional assessment has now become the gold standard in the field of probiotic research.

During preliminary studies on Tibetan kefir, we isolated a strain of *P. pentosaceus*. To our knowledge, systematic genomic and phenotypic characterization of *P. pentosaceus* isolated from Tibetan kefir grains remains limited. To address this gap, we adopted a dual strategy combining physiological characterization with genomic analysis to evaluate the safety profile and probiotic potential of strain K6. Preliminary morphological and biochemical characterization and 16S rRNA gene sequencing were first conducted to identify the strain. Whole-genome sequencing and bioinformatics analysis were then used for genotypic safety assessment and mining of probiotic-associated genes, while *in vitro* assays were applied to evaluate safety-related and probiotic-related properties. This integrated assessment provides genomic and phenotypic evidence for the further evaluation of the Tibetan kefir-derived *P. pentosaceus* strain as a potential probiotic and food biopreservation candidate.

## 2 Materials and methods

### 2.1 Identification and preservation of strain K6

Strain K6 was originally obtained from Tibetan kefir grains, purified and preserved by Food Microbiology Laboratory of Huainan Normal University. The glycerol-preserved K6 was activated in MRS medium and subcultured three times at 37 °C for 24 h. The activated K6 strain was characterized based on colony morphology, Gram staining, and ultrastructural characterization using scanning electron microscopy (SEM). The biochemical identification was performed using VITEK-2 compact system. For molecular identification, complete genomic DNA extraction was performed, followed by PCR-based amplification and sequencing targeting the 16S rRNA gene. A phylogenetic tree was subsequently generated from the sequencing results via the neighbor-joining method.

### 2.2 Whole genome sequencing and bioinformatic analysis

The genomic DNA of strain K6 was extracted and sequenced by Lingen Biotechnology Co., Ltd. (Shanghai, China), utilizing a hybrid approach combining the Illumina NovaSeq and PacBio RS sequencing platforms. Coding sequences (CDS) were predicted using GeneMarkS-2 (Lomsadze et al., 2018) and functionally annotated against the following databases including COG, GO, KEGG, CAZy, CARD, and VFDB (Ruiz-Ramírez et al., 2023). Secondary metabolite biosynthetic gene clusters (BGCs) were identified using antiSMASH 8.0^1^ (Lin et al., 2023).

### 2.3 Phenotypic safety evaluation

#### 2.3.1 Hemolysis test

The hemolysis of K6 was assessed on Columbia sheep blood agar (5%, v/v). Following incubation at 37 °C for 24 h, plates were examined for hemolytic zones. Hemolysis was classified as *α*-hemolysis (partial lysis, greenish zones), *β*-hemolysis (complete lysis, transparent zones), or *γ*-hemolysis (no hemolytic zones) (Khushboo et al., 2023).

#### 2.3.2 Harmful metabolites analysis

Indole test: A 3% (v/v) inoculum of strain K6 was subjected to a 24-h incubation at 37 °C in tryptone broth. Subsequently, a few drops of Kovac’s reagent were added, and the mixture was gently swirled. A positive result was characterized by the appearance of a red surface ring. To corroborate the validity, parallel tests were established using a plain uninoculated broth as negative control and *Escherichia coli* (BNCC336902) as positive control.

Amino decarboxylase activity assay: To determine the amino decarboxylase activity, a 3% (v/v) inoculum of K6 suspension was introduced into the Moeller decarboxylase medium containing either arginine, tyrosine, lysine or histidine. The plates were subjected to a 24-h incubation at 37 °C, after which the decarboxylase activity was determined by observing the color change from yellow to purple. The reliability of this observation was ensured by referencing *E. coli* culture as the positive control and an unsupplemented medium as blank control.

Nitrate reductase assay: Strain K6 was examined for nitrate reductase activity with an inoculum of 3% (v/v) in nitrate broth and incubated at 37 °C for 48 h. After incubation, sulfanilic acid was added followed by *α*-naphthylamine for nitrite detection. The immediate appearance of a red color in the mixture served as positive indicator of reduction to NO₂^−^. In cases without any color change, zinc powder was added to confirm complete reduction. Thereafter, the absence of any color change confirmed the complete reduction of NO₂^−^ (positive), whereas a red coloration suggested incomplete reduction (negative). Throughout the procedure, *E. coli* and uninoculated culture were prepared for positive and negative controls, respectively.

#### 2.3.3 Antibiotic susceptibility testing

To assess the antibiotic susceptibility of strain K6, the Kirby-Bauer disk diffusion test was employed in accordance with CLSI guidelines (Zhang et al., 2025). A K6 suspension (OD_600_ = 0.2 ± 0.05) was subjected to MRS agar plates using sterile swabs. Ten antibiotic discs which are commercially available were aseptically placed on the surface of the medium. The zones of inhibition were measured in mm after incubating at 37 °C for 48 h.

### 2.4 Phenotypic probiotic evaluation

#### 2.4.1 Acid tolerance

An inoculum of K6 (1%, v/v) was added into MRS broth with pH pre-adjusted to 2.0, 3.0 or 6.5 (control) using sterile 1 M HCl. The samples were maintained at 37 °C, and 1 mL aliquots of the culture were sampled separately at 1 h and 2 h to determine viable K6 counts using the plate-counting method. The survival rate was calculated following the method of Diguță et al. (2020) using Equation 1:

$$\text{Strain survival rate}\%=\frac{\mathrm{lg}N_{1}}{\mathrm{lg}N_{0}}\times 100\%$$ (1)

where *N*₁ and *N*₀ represent the viable cell count (CFU/mL) of treatment and control group.

#### 2.4.2 Bile salt resistance

Strain K6 (1%, v/v) was inoculated into MRS broth supplemented with 0.1, 0.3% or 0.5% (w/v) bovine bile salts to assess the bile salt tolerance. Following static incubation at 37 °C, aliquots were collected at 0 h and 3 h, and the viable cell counts were determined using the plate count method. MRS broth without bile salt supplementation served as the control group. Survival rates were also calculated using Equation 1.

#### 2.4.3 Survival of K6 under simulated *in vitro* digestion

To simulate the digestion process, 1 mL of K6 suspension with an *OD*_600_ value of 0.6 ± 0.05 was added to 9 mL of simulated saliva (PH1843, Phygene), thoroughly mixed, and then incubated at 37 °C for 10 min. Subsequently, 1 mL of the oral digest was transferred to 9 mL of simulated gastric fluid (PH1840, Phygene) and incubated at the same temperature for 2 h. Finally, 1 mL of the gastric digestate was added to 9 mL of simulated intestinal fluid (PH1841, Phygene) and incubated at the same temperature for 3 h (Peng et al., 2023). Samples (1 mL) were collected at the end of each digestion phase. The number of viable K6 was determined by the plate count method, and the survival rate was calculated using Equation 1. A mixture of K6 suspension and physiological saline served as the control.

#### 2.4.4 Auto-aggregation ability

The auto-aggregation ability of K6 was determined based on the method of Kokwe et al. (2025) with minor modifications. Briefly, a cell suspension in physiological saline was adjusted to an initial absorbance (*A_0_*) of 0.5 ± 0.05 at 600 nm, and incubated statically at 37 °C to allow for cell sedimentation. The absorbance of the supernatant (*A_i_*) was then measured at 600 nm at intervals of 0, 12, 24, and 48 h. The auto-aggregation percentage was calculated using Equation 2:


$$\text{Auto}-\text{aggregation}\%=\frac{A_{0}-A_{i}}{A_{0}}\times 100\%$$ (2)

#### 2.4.5 Co-aggregation ability

The co-aggregation ability of K6 was evaluated using the method described by Osmanagaoglu et al. (2010) with minor modifications. Equal volumes of the K6 suspension and each of the six pathogenic strain suspensions including *Candida albicans* (BNCC186382)*, Enterococcus faecium* (CICC2252)*, Listeria monocytogenes* (BNCC185986)*, Pseudomonas aeruginosa* (BNCC340634)*, Staphylococcus aureus* (ATCC33591) and *E. coli* (BNCC336902) were mixed. The absorbance of the mixtures was measured at 600 nm immediately and after 4 h of static incubation at 37 °C. The co-aggregation capacity of K6 was then calculated according to Equation 3:


$$\text{Coaggregation}\%=1-\frac{A_{\mathrm{mix}}}{(A_{\text{path}}+A_{K6})/2}\times 100\%$$ (3)

where *A_path_* represents the initial absorbance of pathogenic bacterial suspension, *A_K6_* represents the initial absorbance of K6, and *A_mix_* is the absorbance of mixed culture supernatant after co-aggregation.

#### 2.4.6 Hydrophobicity

The cell surface hydrophobicity of K6 was assessed using a modified version of the method described by Peng et al. (2023). Briefly, a 3 mL aliquot of cell suspension (initial *OD*₆₀₀ = *A₀*) was mixed with 3 mL of xylene or ethyl acetate and vortexed for 30 s. The mixture was allowed to stand for 30 min at 37 °C to ensure complete phase separation. The *OD*₆₀₀ of the aqueous phase was then measured (*A_i_*). The hydrophobicity was calculated using Equation 4:


$$\text{Hydrophobicity}\%=\frac{A_{0}-A_{i}}{A_{0}}\times 100\%$$ (4)

#### 2.4.7 Antibacterial activity assessment

The antibacterial activity of strain K6 was determined using the agar well diffusion assay. To prepare the cell-free supernatant (CFS), K6 was inoculated (2%, v/v) into MRS broth and maintained at 37 °C for 24 h, followed by centrifugation (10, 000 × g, 10 min, 4 °C) to collect the CFS.

The antimicrobial activity of the CFS was evaluated against the same six indicator strains used in the co-aggregation assay. Suspensions of the indicator pathogens were spread evenly onto the surface of agar plates using sterile cotton swabs. After the inoculum was fully absorbed, three sterile Oxford cups were placed on each plate, and 100 μL of K6 CFS was added to each cup. The plates were pre-incubated at 4 °C for 2 h to allow for diffusion, followed by incubation at the optimal temperature for each pathogen for 24–48 h. The antibacterial activity was quantified by measuring the diameters of the inhibition zones (mm).

### 2.5 Statistical analysis

All experiments were performed in triplicate, and data are presented as the mean ± standard deviation (SD). Statistical analyses were performed with GraphPad Prism 9.5.0 software, employing two-way ANOVA, one-way ANOVA followed by Duncan’s multiple range test, and independent Student’s *t*-test. Statistical significance was defined as *p* < 0.05.

## 3 Results and discussion

### 3.1 Identification and preservation of strain K6

**Figure 1:**
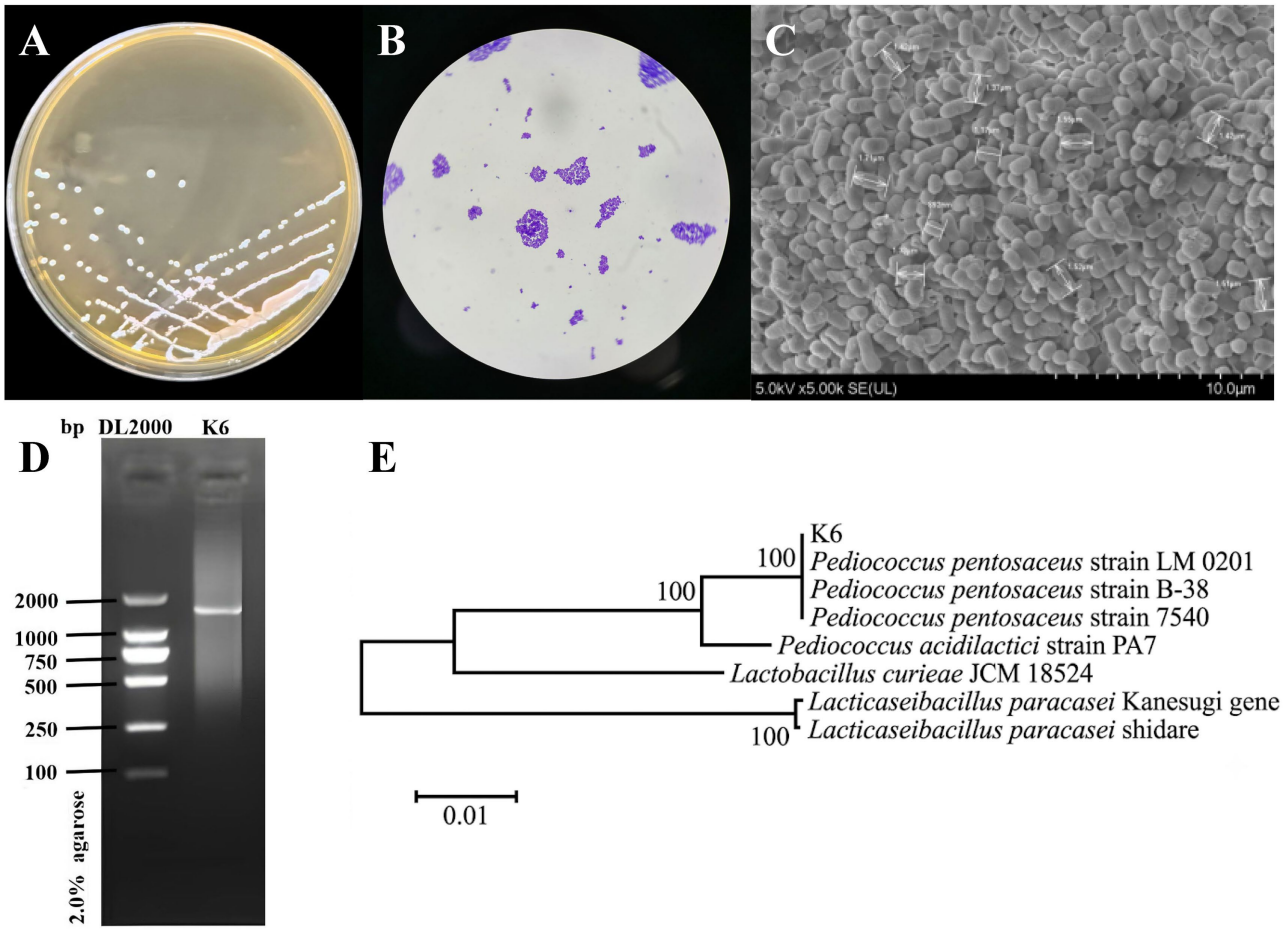
Identification of strain K6. **(A)** Colony morphology, **(B)** Gram staining (1,000×), **(C)** Scanning electron microscopy image (5,000×), **(D)** Agarose gel electrophoresis of amplified 16S rRNA gene, **(E)** Phylogenetic tree based on the 16S rRNA sequences.

The colonies of strain K6 were milky white, circular with a smooth margin, moist and convex (Figure 1A). Gram staining of strain K6 (1, 000 ×) revealed spherical, purple-stained cells consistent with Gram-positive bacteria (Figure 1B). SEM imaging (5, 000 ×) showed ellipsoidal cells with smooth surfaces, measuring 1–2 μm in length and arranged in pairs or clusters (Figure 1C). Based on physiological and biochemical identification, strain K6 was preliminarily identified as *Pediococcus pentosaceus*, and the results are shown in Table 1.

**Table 1:** Physiological and biochemical characteristics of strain K6.

Physiological and biochemical identification indicators	Results	Physiological and biochemical identification indicators	Results
Esculin	+	Salicin	+
Xylose	−	Mannitol	−
Leucine arylamidase	+	Urea	−
Raffinose	+	Maltose	+
Ribose	−	Lactose	+
D-Trehalose	+	Sucrose	+
Cyclodextrin	−	Gelatin Liquefaction	−
H_2_S production	−	Growth at 15 °C	+
L-Lactate alkalinization	−	Glucose	+
Tyrosine arylamidase	−	Catalase Test	−

+, positive; −, negative.

Figure 1D presents the agarose gel electrophoresis profile of the PCR-amplified 16S rRNA gene from strain K6, revealing an amplicon of approximately 1,500 bp, a size consistent with the expected length of bacterial 16S rRNA genes (Weisburg et al., 1991). Following this, the amplification product was sequenced to obtain the 16S rRNA gene sequence of strain K6, which was subsequently deposited in the NCBI database under accession number PQ756913.1. According to the BLAST analysis, this sequence shared 99% identity with *P. pentosaceus* strains LM 0201, B-38, and 7,540. A phylogenetic tree comprising related species was subsequently constructed using MEGA 5.10 (Figure 1E), further confirming the identity of this isolate as *P. pentosaceus* and designating it as K6. The strain *P. pentosaceus* K6 has been deposited in the China General Microbiological Culture Collection Center (CGMCC) under the accession number CGMCC 1.65024.

### 3.2 Genomic features of *P. pentosaceus* K6

**Figure 2:**
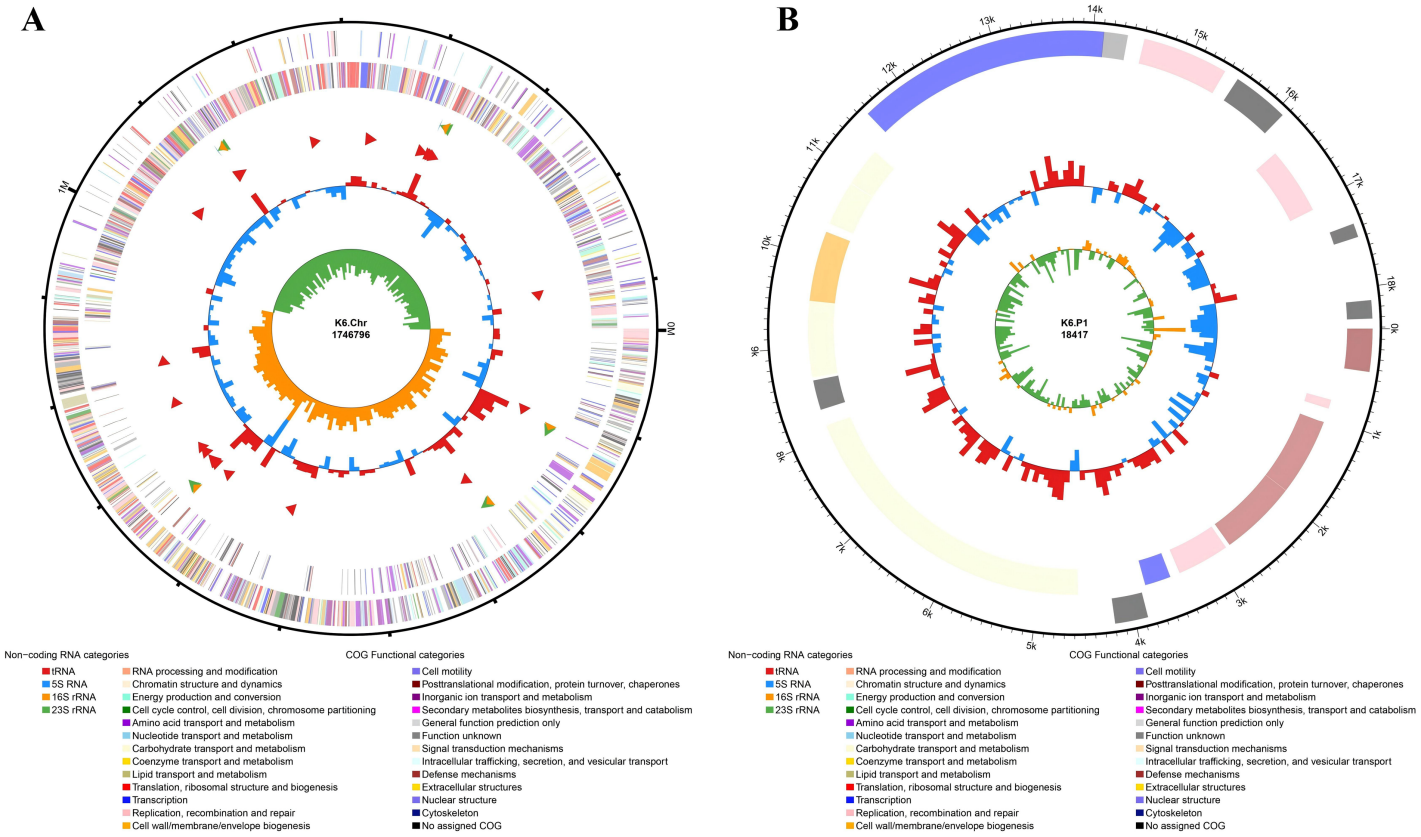
Circular genome map of *P. pentosaceus* K6. **(A)** Chromosome, **(B)** Plasmid.

The genome of *P. pentosaceus* K6 comprises a circular chromosome of 1,746,796 bp (37.4% GC) and a circular plasmid of 18,417 bp (37.96% GC) (Figure 2). Coding gene prediction using GeneMarkS-2 identified a total of 1,779 CDSs, including 20 on the plasmid and 1,759 on the chromosome, along with 56 tRNAs and 15 rRNAs. The predicted CDS regions had an average GC content of approximately 37.93%. The whole-genome sequence data have been deposited in the GenBank database under the BioProject accession number PRJNA1218936.

As previously reported, *P. pentosaceus* genome typically spans 1.7–2.11 Mb, with the number of predicted CDS ranging from 1,692 to 2,170 per genome (Megur et al., 2025). The genome size and CDS count of strain K6 fall well within the normal range reported for *P. pentosaceus* strains. The compact genome of K6 may be attributed to its long-term adaptation to the nutrient-rich kefir matrix (Chaichana et al., 2025), in which K6 did not need to synthesize all essential substances, gradually shed redundant genes through long-term evolution, and eventually developed a streamlined genome adapted to kefir.

### 3.3 Phenotypic safety assessment of *P. pentosaceus* K6

#### 3.3.1 Hemolytic properties

**Figure 3:**
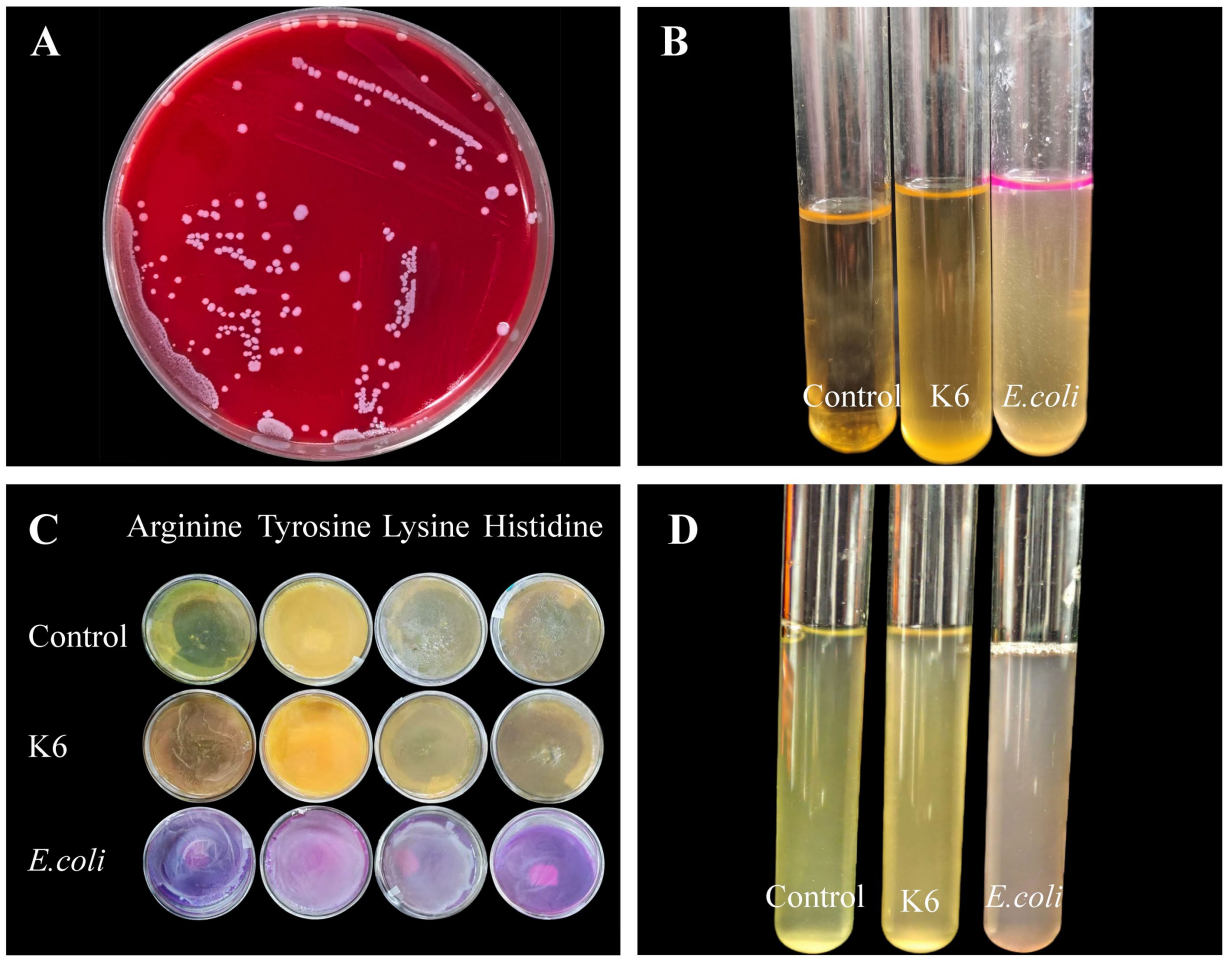
Safety Assessment of *P. pentosaceus* K6. **(A)** Hemolysis test, **(B)** Indole test, **(C)** Amino acid decarboxylase test, **(D)** Nitrate reductase test.

The hemolytic capability of K6 was assessed following FAO safety guidelines. As shown in Figure 3A, the absence of a clear or green zone indicated that *P. pentosaceus* K6 was non-hemolytic, corroborating findings from earlier studies on *P. pentosaceus* (Jiang et al., 2021).

#### 3.3.2 Hazardous metabolite profiling

Indole, characterized by a fecal and putrid odor, serves as a key spoilage indicator of protein putrefaction and potential pathogenicity (Ramadan et al., 2025). The indole test showed an expected rose-red appearance at the surface of the *E. coli* positive control, whereas no color change was observed in either the blank control or strain K6, indicating a negative result (Figure 3B). The lack of indole production reveals that *P. pentosaceus* K6 does not have tryptophanase and is not able to produce indole.

Biogenic amines including histamine, tyramine, agmatine, and cadaverine, are formed by the decarboxylation of amino acids. Excessive accumulation of these substances in fermented foods can pose a threat to health (Gardini et al., 2016). In the amino acid decarboxylase test, the *E. coli* positive control turned purple, whereas both the blank control and *P. pentosaceus* K6 remained yellow, indicating negative amino acid decarboxylase activity (Figure 3C).

In the nitrate reductase test, *P. pentosaceus* K6 yielded a negative result (no color development), consistent with the negative control, whereas the positive control exhibited a pink color (Figure 3D). The fact that K6 cannot convert nitrate to NO₂^−^ is a significant safety advantage, as it would not produce N-nitrosamines during the fermentation of ingredients, such as vegetables and cured meats.

#### 3.3.3 Antibiotic sensitivity assessment

As shown in Table 2, *P. pentosaceus* K6 exhibited high sensitivity to five clinically important antibiotic classes: *β*-lactams, cephalosporins, macrolides, tetracyclines, and chloramphenicol. In contrast, resistance to aminoglycosides, glycopeptides and fluoroquinolones was observed. Extensive research indicates that LAB typically exhibit intrinsic resistance to aminoglycosides and quinolones (Li et al., 2020; Choi et al., 2025). In the genus *Pediococcus*, chromosomally encoded and non-transferable vancomycin resistance is a recognized species-associated trait. In this study, the resistance profile of strain K6 was similar to that of *P. pentosaceus* ZN13P, a strain isolated from Turkish kefir and resistant to vancomycin and clindamycin (Sabir et al., 2010). This phenotypic similarity is consistent with the species-specific genetic behavior of intrinsic resistance in *Pediococcus* (Hummel et al., 2007). Nevertheless, genomic analysis of both chromosomal and plasmid sequences is required to rule out mobile resistance gene elements before application.

**Table 2:** Antimicrobial susceptibility of *P. pentosaceus* K6.

Class	Antibiotics	Disc content	Interpretation criteria/mm			Results	
			S	I	R	Inhibition zone (mm)	Interpretation
β-Lactam	Penicillin	10 units	≥16	13–15	≤12	23.71 ± 1.86c	S
	Amoxicillin	25 μg	≥18	14–17	≤13	23.30 ± 0.83c	S
Aminoglycosides	Streptomycin	10 μg	≥15	12–14	≤11	7.66 ± 1.60d	R
	Gentamicin	10 μg	≥15	13–14	≤12	7.26 ± 0.02d	R
Glycopeptides	Vancomycin	30 μg	≥17	15–16	≤14	9.63 ± 1.20c	R
Cephalosporins	Cefazolin	30 μg	≥18	15–17	≤14	21.45 ± 1.12c	S
Macrolides	Erythromycin	15 μg	≥23	14–22	≤13	29.02 ± 1.08b	S
Tetracyclines	Tetracycline	30 μg	≥19	15–18	≤14	21.32 ± 0.19c	S
Chloramphenicol	Chloramphenicol	30 μg	≥18	13–17	≤12	32.44 ± 0.54a	S
Fluoroquinolones	Norfloxacin	10 μg	≥17	13–16	≤12	8.34 ± 2.39d	R

R, resistant; I, intermediate; S, susceptible.

### 3.4 Genomic safety assessment of *P. pentosaceus* K6

#### 3.4.1 CARD analysis

**Figure 4:**
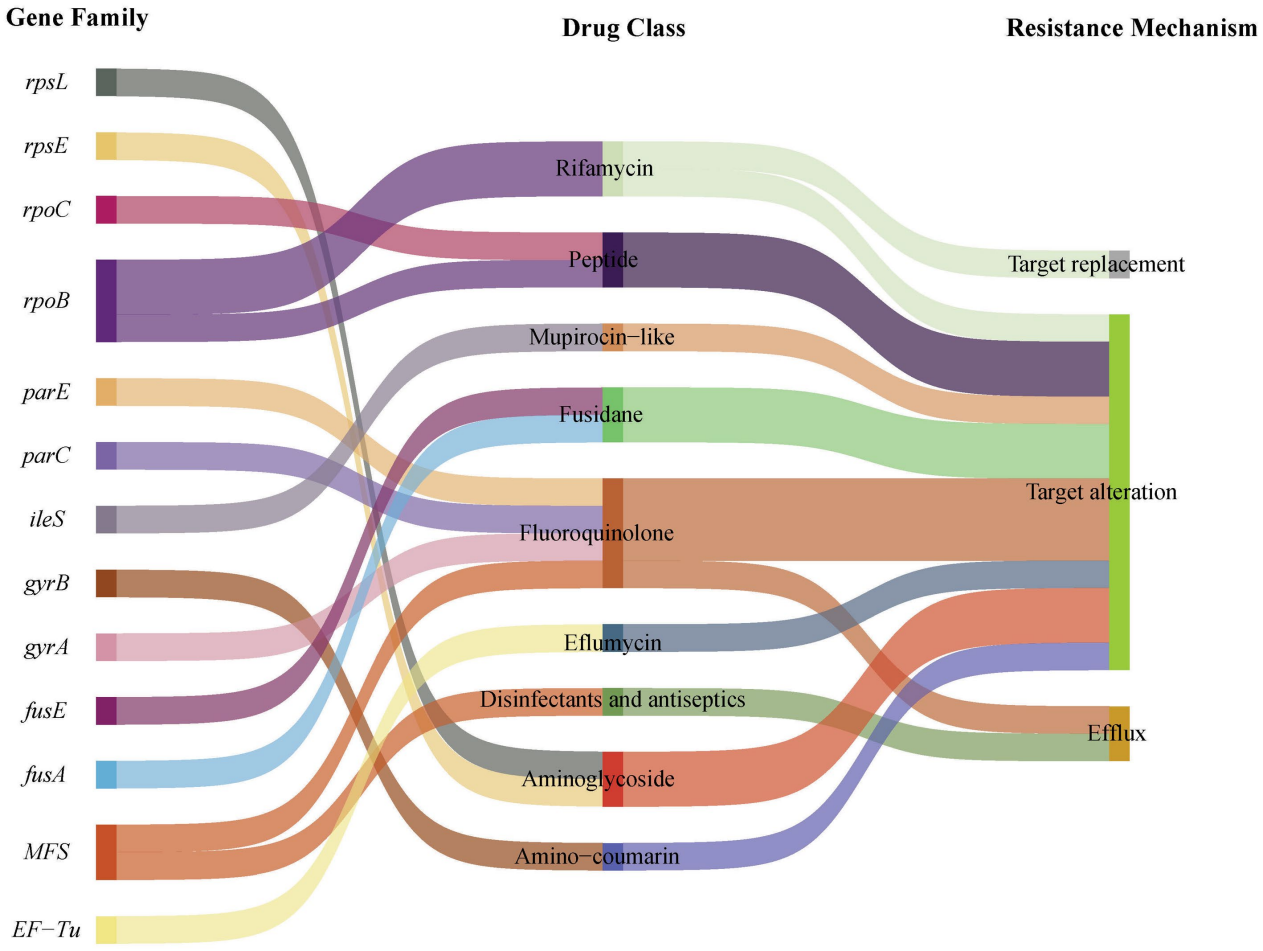
CARD annotation results of *P. pentosaceus* K6.

Given the specific antibiotic sensitivity of *P. pentosaceus* K6, genomic investigation was employed to uncover potential resistance determinants, which demonstrated that the genotype and phenotypic resistance profile of strain K6 were highly consistent. The genes identified by CARD analysis were all chromosomally encoded housekeeping genes, predominantly characterized by alterations in antibiotic targets (Figure 4). Specifically, aminoglycosides resistance was primarily ascribed to naturally low permeability and potential ribosomal protein mutations associated with genes *rpsL* and *rpsE* (Ozma et al., 2024), with no acquired modifying enzyme genes (e.g., *aac*, *ant*, or *aph*) detected (Jaimee and Halami, 2016). Similarly, resistance to fluoroquinolones (e.g., norfloxacin) stemmed from specific point mutations in the chromosomal housekeeping genes *gyrA*, *parC*, or *parE*, rather than the plasmid-encoded quinolone resistance gene *qnr* (Robicsek et al., 2006). CARD analysis also confirmed the absence of high-risk, transferable vancomycin resistance operons, such as *vanA* or *vanB* (Akpınar Kankaya and Tuncer, 2020). Notably, the identified major facilitator superfamily (MFS) efflux pump is a non-specific defense mechanism ubiquitous in LAB, which mediates critical physiological functions and can incidentally confer antibiotic resistance (Drew et al., 2021).

Complementing the chromosomal analysis, plasmid-level safety screening verified the lack of any acquired antibiotic resistance genes. Crucially, the plasmid lacks conjugative transfer gene clusters (*tra/trb*), relaxase-encoding sequences, and the origin of transfer (*oriT*) (Ares-Arroyo et al., 2023). These features indicate that the K6 plasmid is non-mobilizable and lacks self-transmissibility, suggesting a low potential for plasmid-mediated horizontal transfer. Collectively, these findings demonstrate that the observed antibiotic resistance phenotypes of *P. pentosaceus* K6 are most likely attributable to intrinsic chromosomal mechanisms, supporting its biosafety for application.

#### 3.4.2 VFDB analysis

The genome of *P. pentosaceus* K6 was screened against the VFDB database to determine its pathogenic potential. No genes associated with cytolysins or hemolysins (e.g., *cyl*, *hly*, *hla*) were identified in the genome (Kim et al., 2024), and no recognized virulence factors were detected on the K6 plasmid. Meanwhile, six putative virulence factor homologs (*galU*, *clpP*, *eno*, *rfbA*, *rfbB*, and *tuf*) showed limited identity (71.1–81%) to known virulence genes (Table 3) and were functionally annotated as housekeeping genes with core metabolic roles unrelated to pathogenesis. Specifically, the *galU* gene encodes a UDP-glucose pyrophosphorylase involved in polysaccharide synthesis (Lu et al., 2025), while the *eno* gene codes for an enolase possessing both glycolytic function and host adhesion potential (Escobar-Sánchez et al., 2023). The *clpP* gene encodes an intracellular protease involved in stress response and protein homeostasis, and its sequence in strain K6 was identical to those found in well-characterized safe strains M41 (Baig et al., 2021) and ST65ACC (Oliveira et al., 2022). Furthermore, *rfbA* and *rfbB* participate in the synthesis of exopolysaccharides (EPS) vital for colonization and stress tolerance, while *tuf* encodes the universal elongation factor Tu essential for protein synthesis (Ventura et al., 2003). In summary, all identified putative homologs lack pathogenic traits and possess conserved characteristics vital for maintaining normal cellular functions, further confirming the biological safety of strain K6.

**Table 3:** VFDB annotation results for *P. pentosaceus* K6.

GeneID	VFid	Function	Subtype	Identity/%
K6000444	VFG005874	UTP--glucose-1-phosphate uridylyltransferase GalU (involved in cell wall biosynthesis)	*galU*	71.3
K6000456	VFG000077	ATP-dependent Clp protease proteolytic subunit (involved in protein degradation)	*clpP*	71.1
K6000522	VFG005582	Phosphopyruvate hydratase (glycolytic pathway)	*eno*	71.5
K6000619	VFG005898	Glucose-1-phosphate thymidylyltransferase RfbA (polysaccharide assembly)	*rfbA*	73.3
K6000621	VFG006022	dTDP-glucose 4,6-dehydratase (polysaccharide assembly)	*rfbB*	81
K6001083	VFG016490	Elongation factor Tu (involved in protein synthesis)	*tuf*	71.3

### 3.5 In-vitro probiotic evaluation of *P. pentosaceus* K6

#### 3.5.1 Gastrointestinal tract tolerance

**Figure 5:**
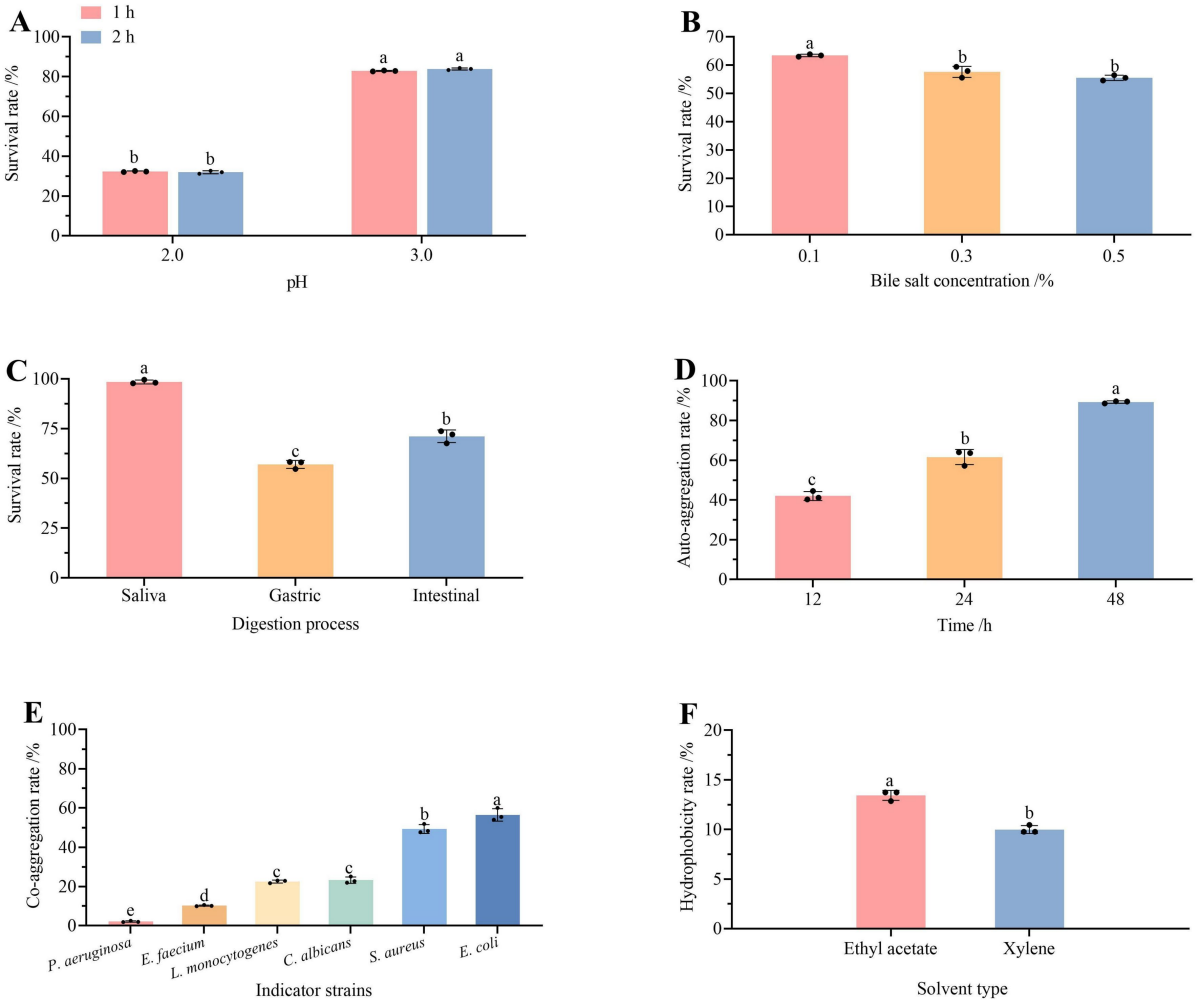
Evaluation of probiotic properties of *P. pentosaceus* K6. **(A)** Survival rate under different acidic conditions, **(B)** Survival rate at varying bile salt concentrations, **(C)** Survival rate under simulated in vitro digestion, **(D)** Auto-aggregation capacity, (E) Co-aggregation capacity, **(F)** Cell surface hydrophobicity. Data were analyzed by two-way ANOVA **(A)**, one-way ANOVA with Duncan’s test **(B–E)**, and Student’s *t*-test (F). Different lowercase letters above the bars indicate statistically significant differences (*p* < 0.05).

Acid tolerance is a fundamental prerequisite for probiotic bacteria because they must endure the gastric acidity before reaching the intestine. As shown in Figure 5A, strain K6 retained 83.83% survival after 2 h at pH 3.0, whereas viability decreased to 31.92% after incubation at pH 2.0 for the same duration. Although survival decreased at pH 2.0, the high viability maintained at pH 3.0 is of great physiological significance, as food intake typically elevates gastric pH to approximately 3.0 (Mennah-Govela et al., 2019). Therefore, it is evident that *P. pentosaceus* K6 demonstrates promising potential for gastric survival and subsequent colonization in the intestine.

As illustrated in Figure 5B, with increasing bile salts concentration, the survival rate of strain K6 declined significantly. Nevertheless, it maintained viability above 55% even at the highest concentration. Since tolerance to physiological concentration of bile salts (0.3% w/v) is essential for probiotic functionality, the results suggest that K6 has the capacity to withstand the challenging environment of upper gastrointestinal tract.

During simulated gastrointestinal digestion, *P. pentosaceus* K6 exhibited a staged change in survival rate (Figure 5C). The strain retained high viability (98.46%) during the simulated salivary phase. After exposure to simulated gastric fluid for 2 h, viability dropped to 57.01%, followed by partial recovery during the 3 h simulated intestinal phase, with a final survival rate of 71.16%. These findings are consistent with reports on *P. pentosaceus* P107 (Blumberg et al., 2025) and notably exceeded the cumulative survival reported for several *Lactiplantibacillus plantarum* strains (29.1–44.2%) under similar conditions (Ruiz-Ramírez et al., 2023). The final viability of *P. pentosaceus* K6 (about 10^7^ CFU/mL) supports its further evaluation for oral delivery. The multi-stress tolerance observed in K6 may be related to long-term adaptation to the kefir matrix, which helps to reinforce specific metabolic pathways that support environmental stress resistance(Huang et al., 2020; Huo et al., 2022).

#### 3.5.2 Adhesion ability

The interaction between lactic acid bacteria and host intestinal cells is primarily mediated through specific and non-specific adhesion, wherein non-specific adhesion is typically assessed based on hydrophobicity and auto-aggregation ability (Krausova et al., 2019). As illustrated in Figure 5D, the auto-aggregation rate increased significantly over time, which attained 41.99% at 12 h and ultimately peaked at 89.30% at 48 h. Overall, the auto-aggregation capabilities of K6 all exceeded 60% after incubating for 24 h, indicating its good adhesion ability.

The co-aggregation efficacy of *P. pentosaceus* K6 showed significant statistical differences (*p* < 0.05) in strain-specificity (Figure 5E). In particular, strain K6 exhibited the strongest co-aggregation ability against two common pathogenic bacteria in raw milk, *E. coli* and *S. aureus*, with co-aggregation rates of 56.49 and 49.27%, respectively. Notably, strain K6 exhibited a higher co-aggregation capacity with *E. coli* compared to kefir-derived strain *P. pentosaceus* K44 (22%) (Sabir et al., 2010) and *P. pentosaceus* OZF (12.99%) isolated from human breast milk (Osmanagaoglu et al., 2010).

The cell surface hydrophobicity of K6 varied significantly with solvent type (*p* < 0.05), exhibiting 13.44% in ethyl acetate and 9.99% in xylene (Figure 5F). These results indicated that strain K6 exhibited a low hydrophobicity and was overall hydrophilic. The hydrophobicity of bacterial surfaces depends largely on the nature of the surface chemical constituents. Typically, the presence of polysaccharides tends to result in lower hydrophobicity, whereas the enrichment of glycoproteins is likely to impart stronger hydrophobic characteristics (Osmanagaoglu et al., 2010). In our study, the low hydrophobicity of strain K6 may be associated with the production of exopolysaccharides, as genes (such as *rfbA* and *rfbB*) involved in EPS synthesis were identified by genomic analysis. However, it is worth noting that factors such as origin, strain specificity, growth age, surface chemical microenvironment, and even the nutrient composition of culture medium can also exert significant influence on hydrophobicity, thereby leading to substantial variations in hydrophobic potential (Zommiti et al., 2018).

#### 3.5.3 Antimicrobial activity

The CFS of *P. pentosaceus* K6 inhibited both Gram-positive and Gram-negative bacteria but was ineffective against *C. albicans* (Table 4). K6 showed the strongest inhibition against *L. monocytogenes*, with an inhibition zone of 29.83 ± 0.06 mm, followed by moderate activity against *P. aeruginosa* (18.66 ± 0.73 mm) and *S. aureus* (17.21 ± 0.31 mm), and the minimal inhibition against both *E. coli* and *E. faecium* (approximately 10.00 mm for each). *L. monocytogenes* is a common foodborne pathogen typically found in ready-to-eat foods such as raw milk and processed meat products, and is renowned for its exceptional adaptability and antibiotic resistance (Rajalingam and Van Haute, 2025). Compared with reported *P. pentosaceus* strains from different food-related matrices, the inhibition zone of K6 against *L. monocytogenes* was larger than that of the marine strain E3 (13–24 mm) (Zaghloul and Halfawy, 2024), the freshwater-fish-derived strain 4I1 (18.5 mm) (Bajpai et al., 2016), the optimized dadih isolated strain 2,397 (12.3 mm) (Pato et al., 2021), the kimchi-derived strain T1 (>6.5 mm) (Jang et al., 2014) and the pickle strain 05–10 (15–25 mm) (Huang et al., 2009). Due to varying evaluation standards, distinct indicator strains, and diverse supernatant processing methods, these reported values are presented as a general guide rather than absolute quantitative metrics. Overall, our results indicate that K6 is a promising candidate for biopreservation of dairy and fermented food products.

**Table 4:** Antibacterial activity of *P. pentosaceus* K6-CFS against pathogens.

Group	Species name	Inhibition zone (mm)
G-	*Escherichia coli*	10.00 ± 0.14^d^
	*Pseudomonas aeruginosa*	18.66 ± 0.73^b^
G+	*Staphylococcus aureus*	17.21 ± 0.31^c^
	*Listeria monocytogenes*	29.83 ± 0.06^a^
	*Enterococcus faecium*	10.00 ± 0.10^d^
Yeast	*Candida albicans*	—

### 3.6 Genomic evaluation of the probiotic potential of *P. pentosaceus* K6

#### 3.6.1 COG analysis

**Figure 6:**
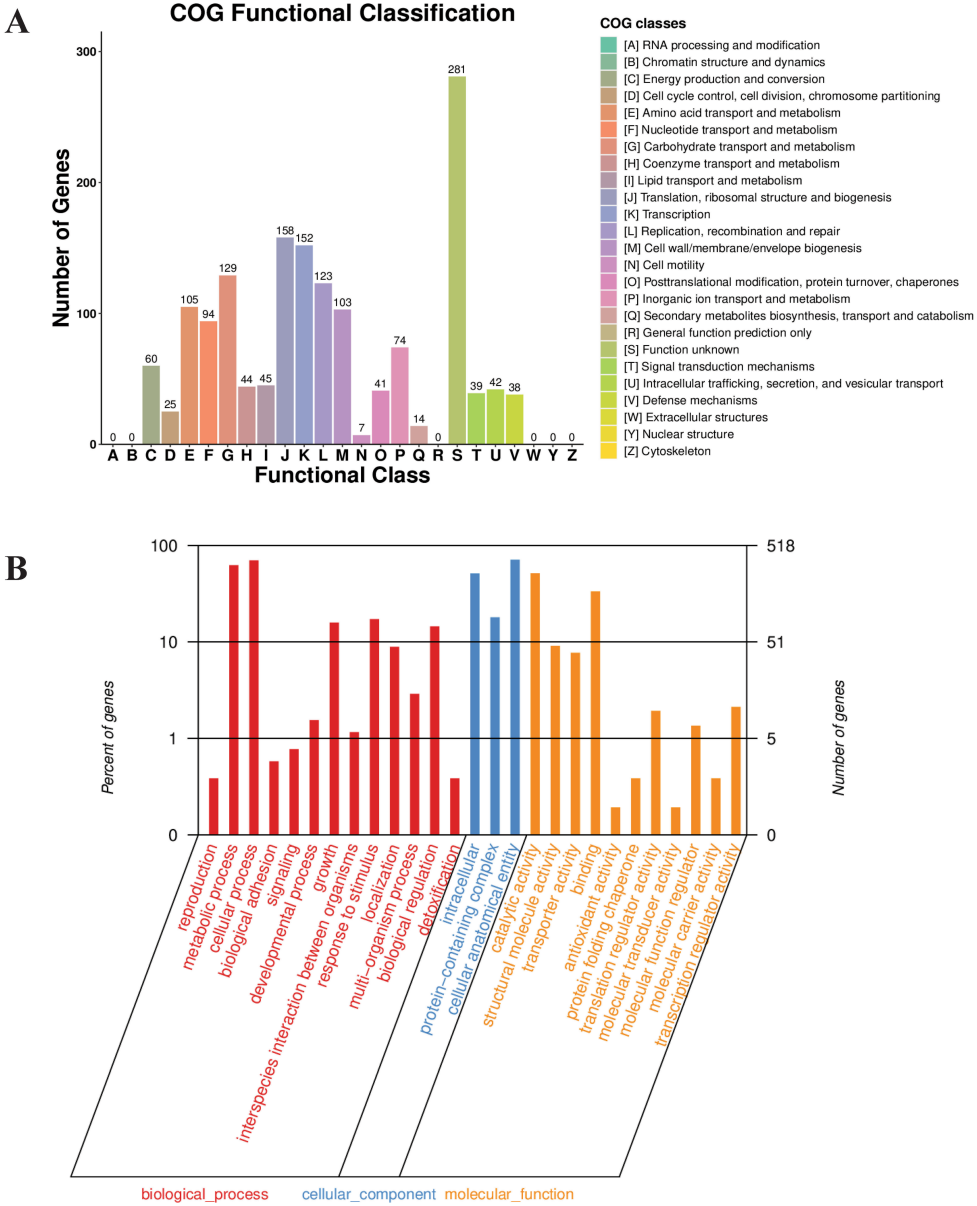
COG and GO annotation analysis of the *P. pentosaceus* K6 genome. **(A)** COG functional classification, **(B)** GO term annotation.

Functional genes associated with the metabolic activity of *P. pentosaceus* K6 were identified via the eggNOG database. A total of 1,574 COG functional units were distributed across 19 major categories (Figure 6A). Excluding the function-unknown category (S, 281 genes), the distribution revealed a robust genetic basis for cellular maintenance and metabolic activities. Notably, genes associated with translation and ribosome biogenesis (J, 158 genes), transcription (K, 152 genes), and replication, recombination and repair (L, 123 genes) were highly enriched, enabling strain K6 to achieve rapid proliferation and an effective response to stressful environments (Rahman et al., 2024). In terms of metabolism, genes for the transport and metabolism of amino acid (E, 129 genes), nucleotide (F, 94 genes), and inorganic ions (P, 74 genes) were significantly enriched, enhancing nutrient utilization and survival in the kefir matrix (Huang et al., 2025). Furthermore, substantial gene clusters associated with cell wall/membrane/envelope biogenesis (M, 103 genes), intracellular trafficking, secretion and vesicular transport (U, 42 genes), and signal transduction (T, 39 genes) were also identified, indicating that strain K6 can effectively maintain structural integrity and withstand environmental stresses, providing a genetic basis for its strong multi-tolerance. The presence of genes related to defense mechanisms (V, 38 genes) further suggested its potential for environmental adaptability and stress resistance.

#### 3.6.2 Functional annotation by GO

The predicted genes of *P. pentosaceus* K6 were annotated against the GO database to characterize its functional profile. Metabolic and cellular process were the dominant terms within the biological process category (Figure 6B), consistent with the high enrichment of catalytic activity and binding in the molecular function category. Genes associated with growth, response to stimulus, and biological regulation also accounted for a significant proportion, demonstrating that strain K6 possesses capabilities for growth regulation, stress response and environmental adaptation. These findings provide genetic evidence for its potent *in vitro* properties including acid tolerance, bile salt resistance, and gastrointestinal transit tolerance (Huang et al., 2025). Regarding cellular components, genes were primarily mapped to intracellular and cellular anatomical entity, and protein-containing complexes, which conformed to the general characteristics of lactic acid bacteria cell structure. The molecular function category exhibited a distribution pattern dominated by catalytic and binding activities, indicating that *P. pentosaceus* K6 encodes a large number of enzymes and binding proteins, which offers a molecular basis for its metabolic diversity, substrate utilization capabilities, and potential extracellular interactions.

#### 3.6.3 KEGG analysis

**Figure 7:**
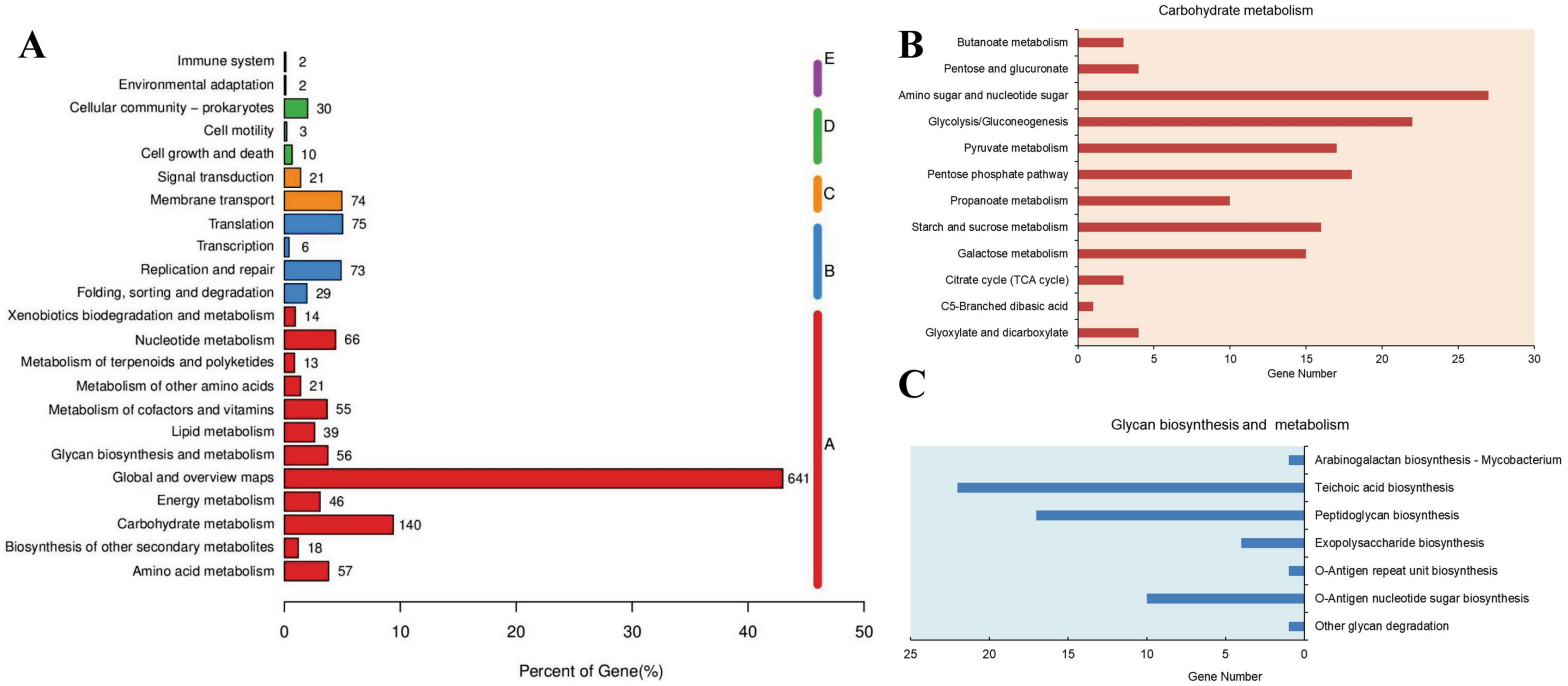
KEGG pathway annotation and analysis of major metabolic functions of *P. pentosaceus* K6 genome. **(A)** KEGG pathway annotation, **(B)** Carbohydrate metabolism, **(C)** Glycan biosynthesis and metabolism.

To fully understand the metabolic potential and physiological capacity of *P. pentosaceus* K6, a system-level analysis of gene functions was conducted using the KEGG pathway database. Figure 7A indicates that metabolism (A) targets the maximum number of pathways with the richest functionality. The predominance of global and overview maps (641 genes) demonstrated that strain K6 had well-developed metabolic functions. Specifically, substantial amounts of carbohydrate metabolism pathways (140 genes) suggested its powerful capability of carbohydrate utilization and prebiotic degradation. It can be seen from Figure 7B that the most highly enriched pathways were amino sugar and nucleotide sugar (27 genes), glycolysis/gluconeogenesis (22 genes), pentose phosphate pathway (18 genes), pyruvate metabolism (17 genes), starch and sucrose metabolism (16 genes) and galactose metabolism (15 genes). Amino and nucleotide sugars function as precursors in the biosynthesis of EPS (Lu et al., 2024). The predominance of genes involved in these two metabolisms indicated the robust potential of *P. pentosaceus* K6 for EPS production (Qi et al., 2021). Enrichment of the pentose phosphate pathway corroborated the species-specific characteristic of K6 in pentose metabolism. Notably, the enrichment of the galactose metabolic pathway suggested its proficiency in degrading lactose derivatives, thereby ensuring its robust growth in kefir-like dairy habitats.

In addition to multiple carbohydrate metabolism pathways, strain K6 possessed abundant pathways for nucleotide, amino acid, cofactor and vitamin metabolism, as well as glycan synthesis and metabolism (Figures 7A,C), demonstrating its robust capacity to synthesize substances necessary for growth, as well as its potential to provide nutrients to the host (Das et al., 2019). Particularly, enriched glycan biosynthesis and cellular community pathways further reinforced the EPS production and biofilm formation capacity critical for mucosal adhesion and colonization, providing genetic evidence for its robust tolerance and potential probiotic properties (Lu et al., 2024). It is well established that kefir grains contain a complex microbial community of various bacteria and yeasts living in symbiosis (Sabir et al., 2010). The cellular community pathways revealed the genetic foundation for the integration of strain K6 into the symbiotic kefir community, as well as coexistence and co-fermentation with other microorganisms. Moreover, enriched membrane transport and signal transduction pathways promoted environmental perception and rapid response, while the translation, replication, and repair pathways highlighted its capacity for rapid proliferation and stable inheritance. Collectively, KEGG analysis demonstrates that *P. pentosaceus* K6 possesses well-developed functions for reproduction, metabolism, membrane transport, nutrient synthesis, biofilm formation, and quorum sensing, manifesting its strong probiotic potential and promising application prospects.

#### 3.6.4 Functional annotation by CAZyme

**Figure 8:**
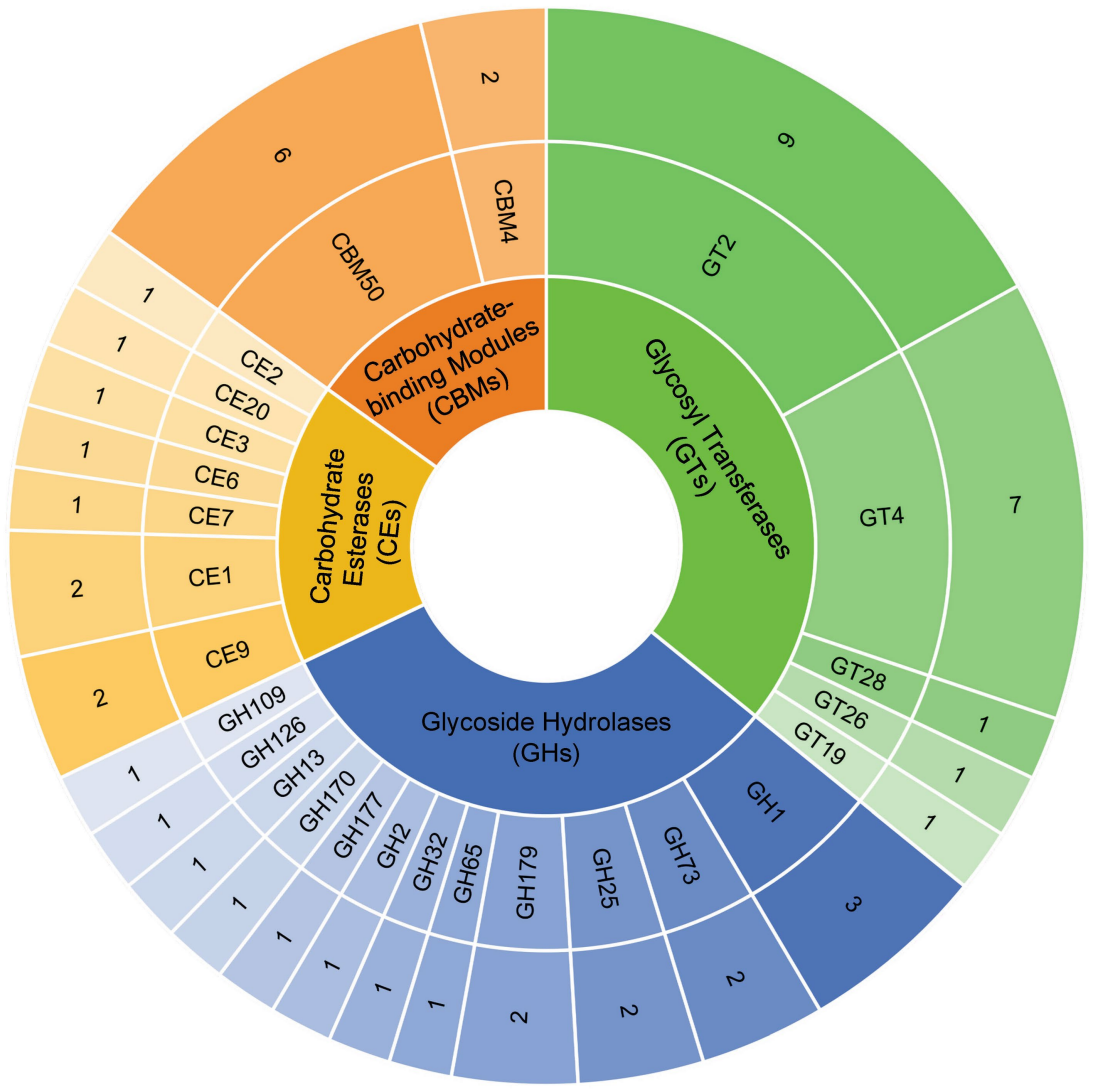
Distribution of CAZymes encoded by *P. pentosaceus* K6 genome.

To further uncover the potential carbohydrate metabolic capabilities of *P. pentosaceus* K6, we annotated its genome against the CAZyme database. CAZymes are critical for the metabolism of complex carbohydrates and glycoconjugates, which comprise six functional categories, including glycoside hydrolases (GHs), glycosyltransferases (GTs), polysaccharide lyases (PLs), carbohydrate esterases (CEs), carbohydrate-binding modules (CBMs), and auxiliary activities (AAs) (Shi et al., 2026). According to Figure 8, a total of 56 genes were identified across four families, among which GTs constituted the predominant family with 22 genes, followed by GHs with 17 genes. CEs and CBMs were represented by 9 and 8 genes, respectively. The diverse groups and families of CAZymes in *P. pentosaceus* K6 demonstrated its strong capability for carbohydrate utilization and energy generation. The predominant GT2 and GT4 enzymes, along with abundant nucleotide sugar pathways (Figure 7B), conclusively demonstrated a strong capacity for EPS synthesis, which facilitate the formation of biofilms within kefir grains, the maintenance of community stability, and also the textural enhancement of kefir (Angelin and Kavitha, 2020). The GH1 and GH2 families typically contain *β*-galactosidase that can hydrolyze lactose into glucose and galactose (Kalathinathan et al., 2023), which may subsequently participate in the highly enriched glycolysis (22 genes) and galactose metabolism (15 genes) pathways for further conversion. These findings indicated that strain K6 possessed robust lactose metabolic capabilities, aligning perfectly with its highly enriched galactose metabolism pathways. As carbohydrate-binding modules within various enzymes, the CBM50 family primarily participates in the degradation of peptidoglycans and chitin, whereas CBM4 family predominantly mediate breakdown of complex polysaccharides such as cellulose, xylan, and glucan. Moreover, a wide variety of other GHs and CEs including GH13, GH32 (plasmid-encoded), CE1, CE9, as well as the enriched starch and sucrose metabolism pathways (Figure 7B) further demonstrated the capacity for the utilization of plant-derived carbohydrates (Fuhren et al., 2020). Collectively, the annotated CAZymes genes in *P. pentosaceus* K6 provided genetic evidence for the potential capability of EPS synthesis and carbohydrate utilization, suggesting its potential probiotic functions and fermentation applications.

#### 3.6.5 Functional annotation by antiSMASH

**Figure 9:**
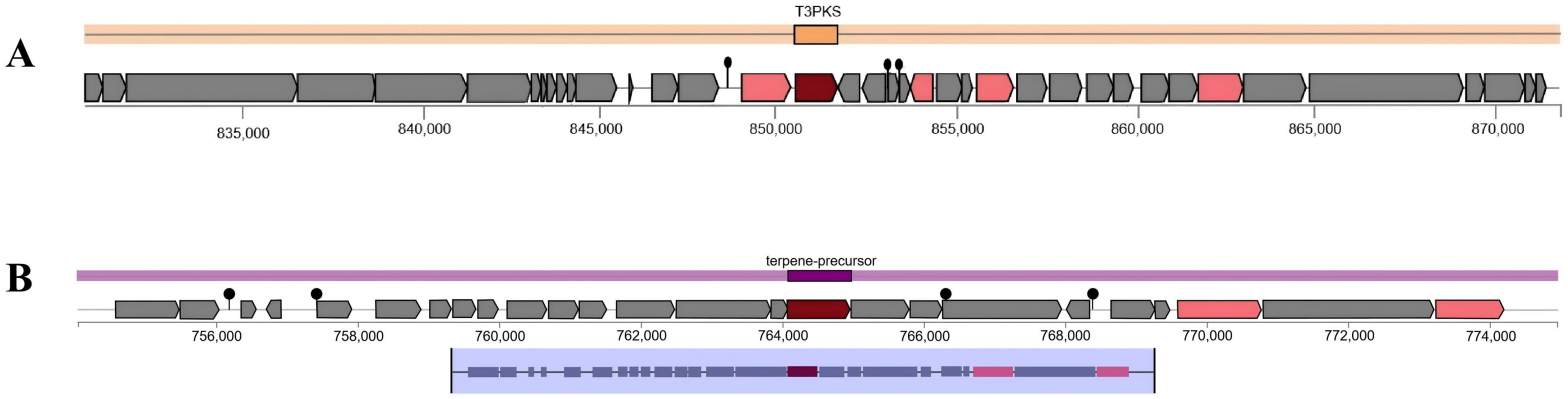
Annotation of T3PKS and terpene gene clusters in *P. pentosaceus* K6 via antiSMASH. **(A)** type III polyketide synthase (T3PKS) cluster, **(B)** terpene-precursor cluster.

Genome mining using the antiSMASH database identified two putative secondary metabolite BGCs, namely a type III polyketide synthase (T3PKS) cluster and a terpene-precursor cluster (Figure 9), suggesting the genetic potential of *P. pentosaceus* K6 to synthesize polyketide- or terpene-related metabolites. This prediction is consistent with the KEGG annotation of pathways related to terpene and polyketide metabolism and secondary metabolites biosynthesis (Figure 7A). T3PKSs can catalyze the biosynthesis of amphipathic polyketides that may affect cell membrane and quorum sensing (Lim et al., 2016), while terpenoids can interfere with or disrupt cell membranes (Ergüden, 2021). Thus, the predicted clusters provide mechanistically plausible candidates potentially associated with the potent anti-*Listeria* activity of the K6 CFS.

At present, the specific bacteriostatic compounds in the CFS of K6 have not yet been chemically isolated or identified, and the antibacterial mechanism inferred from genomic gene clusters remains to be further validated. Nevertheless, studies on the antibacterial properties of postbiotics derived from other lactic acid bacteria have provided us with valuable insights. Postbiotics are bioactive components secreted by live bacteria or released following cellular lysis, capable of conferring health benefits to the host. Hossain et al. (2021) demonstrated that postbiotics from two *Lactobacillus* strains exert multidimensional anti-*Listeria* activity by effectively blocking its motility, biofilm assembly, and quorum sensing. Similarly, Shi et al. (2025) found that postbiotics derived from *Ligilactobacillus salivarius* LSA-6 can disrupt the integrity of *L. monocytogenes* cell membranes, triggering the leakage of intracellular contents and ultimately leading to cell death. Furthermore, they confirmed that the characteristic metabolic products of these postbiotics are organic acids and fatty acids. For strain K6, the identification of T3PKS and terpene-precursor BGCs, as well as the pathways for glycan biosynthesis and carbohydrate metabolism suggests a genomic basis for the inhibitory effects mediated by the postbiotics, including organic acids, polyketides, terpenoids, EPS and other potential antibacterial components. In subsequent studies, metabolomic profiling, physicochemical treatments and bioactivity-directed purification will be conducted to fully clarify the antimicrobial mechanism of *P. pentosaceus* K6.

## 4 Conclusion

This work presents a systematic genomic and phenotypic characterization of *P. pentosaceus* K6 isolated from Tibetan kefir grains. Strain K6 demonstrated favorable preliminary safety performance, including negative hemolytic activity, absence of harmful metabolites and high-risk transferable antibiotic resistance determinants. *P. pentosaceus* K6 also exhibited remarkable gastrointestinal tolerance, high adhesion capabilities and notable anti-*Listeria* activity. Abundant pathways for carbohydrate metabolism, glycan metabolism, cofactor and vitamin biosynthesis, CAZymes, and putative T3PKS and terpene-precursor clusters provide hypotheses for its adaptation to fermented food matrices and deepened mechanistic insights. These findings support *P. pentosaceus* K6 as a candidate for further evaluation as a functional starter culture or natural biopreservation agent, highlighting the value of Tibetan kefir as a promising reservoir of beneficial probiotics.

## Data Availability

The datasets presented in this study can be found in online repositories. The names of the repository/repositories and accession number(s) can be found at: https://www.ncbi.nlm.nih.gov/genbank/, PQ756913.1.

## Footnotes

1
https://antismash.secondarymetabolites.org
